# Vibrational spectrum of Granular packings with random matrices

**DOI:** 10.1140/epje/s10189-024-00414-x

**Published:** 2024-03-12

**Authors:** Onuttom Narayan, Harsh Mathur

**Affiliations:** 1 Physics Department, University of California, Santa Cruz, CA 95064 USA; 2 Department of Physics, Case Western Reserve University, Cleveland, OH 44106-7079 USA

## Abstract

**Abstract:**

The vibrational spectrum of granular packings can be used as a signature of the jamming transition, with the density of states at zero frequency becoming nonzero at the transition. It has been proposed previously that the vibrational spectrum of granular packings can be approximately obtained from random matrix theory. Here, we show that the autocorrelation function of the density of states shows good agreement between dynamical numerical simulations of frictionless bead packs near the jamming point and the analytic predictions of the Laguerre orthogonal ensemble of random matrices; there is clear disagreement with the Gaussian orthogonal ensemble, establishing that the Laguerre ensemble correctly reproduces the universal statistical properties of jammed granular matter and excluding the Gaussian orthogonal ensemble. We also present a random lattice model which is a physically motivated variant of the random matrix ensemble. Numerical calculations reveal that this model reproduces the known features of the vibrational density of states of frictionless granular matter, while also retaining the correlation structure seen in the Laguerre random matrix theory.

**Graphic abstract:**

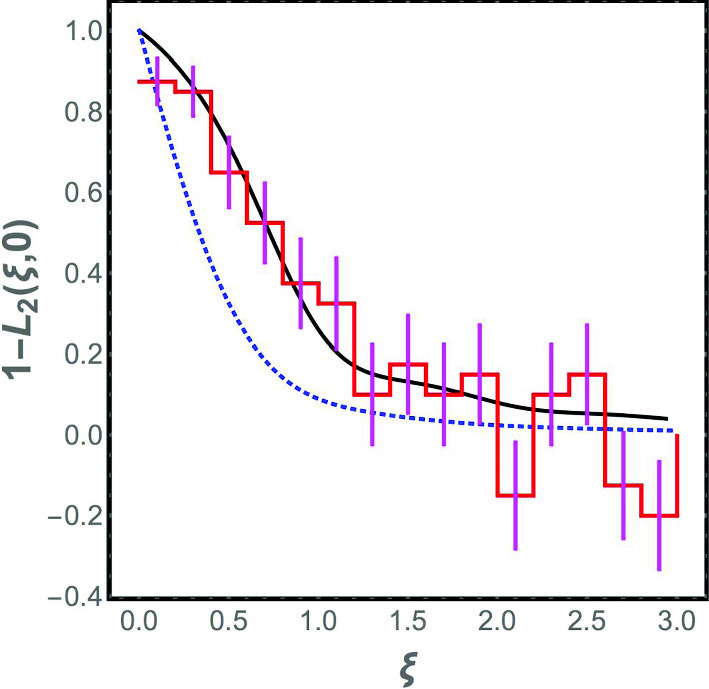

## Introduction

Granular materials are a class of systems which are out of equilibrium and not easy to understand within the framework of standard statistical mechanics. For static assemblies, the distribution of forces [1, 2] and the continuum limit [3] are difficult to obtain. This is because interparticle contacts are very stiff: a slight compression of two particles that are in contact, by an amount that is much less than the interparticle separation, gives rise to large forces. Added complications are caused by the fact that, for noncohesive granular matter, two particles in contact repel each other when they are compressed, but do not attract each other when they are moved away from each other and the contact is broken; that the repulsive force between particles is not a linear function of their compression when the compression is small; [4] and that there are frictional forces between particles, [5] resulting in history dependent forces. The dynamic properties of granular matter are difficult to understand because interparticle collisions are strongly inelastic. If a high density of particles builds up in a region because of random fluctuations, the collision rate and therefore the rate of energy loss increases in the region. This can trap particles in the region, causing the density fluctuations to grow. [6] Experimentally, one observes distinctive phenomena such as force chains and stability against mechanical collapse in very sparse static packings, non-Maxwellian velocity distributions [7–9] and inelastic collapse in dilute granular gases, [10, 11] and shear thinning and shear thickening. [12]

The tendency of flowing granular matter to get ‘jammed’ and stop flowing at low densities is a practical problem that limits the flow rate in the industrial use of granular materials. [13] Remarkably, the transition from a flowing to a jammed state in granular matter, structural glasses, and foams and colloids, can be studied with a unified approach. [14] When the transition occurs at zero temperature and zero shear stress as the density is varied, the transition point is called ‘Point J,’ [15] and is characterized by diverging length scales [16, 17] suggestive of a second-order phase transition. At the same time, other properties of the system change discontinuously at Point J [15] as one would expect at a first-order phase transition.

The density of states for vibrational modes in a granular system is one of the properties that has a signature of the transition at Point J. A jammed granular system has mechanical rigidity. Even though the force between two particles is a nonlinear function of the compression between them, the small deviations from the jammed state (which already has nonzero compression) can be analyzed using a linear model, resulting in normal modes. Extensive numerical simulations [15] on systems at zero temperature and zero shear stress show that the density of states $\rho (\omega )$ as a function of $\omega$ approaches zero linearly as $\omega \rightarrow 0$ if the particle density is greater than the critical density. As the particle density is reduced, the slope of $\rho (\omega )$ at the origin becomes steeper, until at Point J, $\rho (\omega \rightarrow 0) \ne 0.$

In the linearized analysis of vibrational modes, the system can be treated as a network of random springs, with the number of springs decreasing as Point J is approached. It is natural to analyze the problem using random matrices, and see how the resultant density of states evolves near the transition. This has been done [18, 19] using the Laguerre ensemble instead of the Gaussian orthogonal ensemble (GOE) for the random matrices, in accordance with—as discussed in the next section of this paper—the symmetries of the problem. The model yields a broad peak in the density of states that reaches $\omega = 0$ as the transition is approached. However, the model also predicts a gap in the density of states near $\omega = 0$ above the transition, which does not match the numerical results. This is not surprising, because the density of states predicted by random matrix theory is well known to suffer from non-universal effects [20]. For instance, the density of states can be changed at will by varying the assumed distribution of the matrix elements. [21] Instead, the correlations in the density of states and the distribution of level spacings are more reliable indicators of the validity of the random matrix approach [20].

The agreement of the level spacing distribution with the GOE result has been observed elsewhere [22, 23] without reference to the Laguerre ensemble. The distribution of level spacings has been studied for the Laguerre ensemble [24], without comparing to the numerical data for bead packs, but it is unfortunately indistinguishable from the results for the GOE. Taken together, Refs. [22–24] show that the level spacing distribution for bead packs is consistent with random matrix theory, without being able to confirm the appropriate ensemble.

To summarize, the density of states for random matrix ensembles is known to be a non-universal feature; not surprisingly, therefore, the density of states for the simplest version of the Laguerre ensemble shows qualitative differences from the density of states of granular vibrational modes observed in numerical simulations. On the other hand, the level spacing distribution is found to be *too* universal: there is agreement between the Laguerre ensemble and numerical simulations, but there is equally good agreement between the GOE and numerical simulations. Thus, neither of these can be considered as conclusive evidence for the Laguerre ensemble.

In this paper, we therefore turn to the correlations in the density of states predicted by random matrix theory. We consider the correlation function for the Laguerre ensemble, which *differs* from that for the GOE near the low-frequency edge of the allowed range of $\omega$ [25]. By comparing the correlations in the numerically computed vibrational spectrum of frictionless bead packs near the jamming transition to the predictions of the Laguerre ensemble and the GOE, we are able to demonstrate good agreement with the former and to exclude the latter.

In addition, we construct a random lattice model, which is a physically motivated variant of the random matrix ensemble. Although it is not possible to calculate the properties of this model analytically, numerical results reveal that all the qualitative features of $\rho (\omega )$ are reproduced. At the same time, the correlation functions and the level spacing distribution seen in the idealized random matrix theory are not significantly changed. Models that are essentially mean field versions of our model have been studied earlier [26, 27], and differ from our results mainly in the fact that the low-frequency $\rho (\omega ) \sim \omega ^{d-1}$ behavior for a *d*-dimensional system is not seen. The random lattice model is a variation of a model used earlier to study the static properties of free-standing granular piles (with vector forces) [28]; the fact that such similar models yield results in agreement with different experiments increases the credibility of the model.

Other authors have studied variations of random matrix theory: Ref [29] is an early random matrix model for the related problem of the vibrational spectrum of glasses close to the glass transition. Reference [30] studies several different random matrix ensembles and their effect on the structure of eigenmodes with frequencies in the boson peak. Reference [31] uses weighted Laplacian dynamical matrices to reproduce an intermediate regime in $\rho (\omega )$ (between the boson peak and the low-frequency behavior) and $\sim \omega ^4$ scaling of the density of states in this regime. Reference [32] uses a combination of a random and a regular matrix for the dynamical matrix, to eliminate the gap near $\omega = 0.$ Also, Ref. [33] has studied an abstract model that they argue is in the appropriate universality class.

The rest of this paper is organized as follows. Section 2 reviews why the symmetry properties of the systems in which we are interested are in the Laguerre universality class rather than the GOE. Section 3 has the first main result of this paper: that the analytically calculated autocorrelation function for the density of energy levels of the Laguerre orthogonal ensemble agrees with the correlation function from dynamical simulations on bead packs, in the vicinity of Point J, but that this is not true for the correlation function for the Gaussian orthogonal ensemble. Section 4 has the second main result of this paper: that the random lattice model, which retains some spatial correlations discarded by the simple random matrix model, is numerically found to have a similar behavior for the density of energy levels as seen in the molecular dynamics simulations, both at and away from Point J. The energy level correlation function and the level spacings for the random lattice model are also seen to agree with the molecular dynamics results (and the Laguerre ensemble results), in accordance with the expectation that such correlations should only depend on the symmetries of the problem instead of the model details. Thus, the random lattice model retains the points of agreement between the Laguerre ensemble and molecular dynamics, and also successfully reproduces the density of states.

## Laguerre ensembles

We follow the approach of Ref. [18, 19] here. Within linear response, if the particles in a frictionless granular assembly are displaced slightly from their resting positions, their accelerations are of the form $\ddot{x} = -K x,$ where *x* is a *N* component column vector with (a rescaled version of) the displacements of all the particles and *K* is a $N\times N$ matrix called the dynamical matrix [34]. If *d* is the dimensionality of the granular assembly, then each particle has *d* components to its displacement, and *N* is therefore equal to *d* times the number of particles.

The crucial observation [35, 36] is that the connection between accelerations and displacements is a two-step process. Within linear response, each contact between a pair of particles can be represented as a spring that has been precompressed by some amount. Thus, one has a network of springs, with various spring constants. When a particle is displaced, it stretches (or compresses) each spring that it is connected to, by an amount that is equal to the component of its displacement along that spring. Thus, we have

1 $$\begin{aligned} \Delta \ell _\alpha = {\tilde{A}}_{\alpha j} x_j \end{aligned}$$

where $\Delta \ell _\alpha$ is the amount by which the spring labeled $\alpha$ is compressed. Here, $x_j$ is one component of the displacement of one of the particles in the assembly. If the particle is one of the two that is involved in the $\alpha$’th contact, then ${\tilde{A}}_{\alpha j}$ is simply $\cos \theta _{\alpha j},$ where $\theta _{\alpha j}$ is the angle between the direction of the $\alpha$’th spring and the direction of the *j*’th displacement, i.e., $\cos \theta _{\alpha j} x_j$ is the compression of the contact caused by the displacement. If $x_j$ is not associated with either of the particles involved in the $\alpha$’th contact, then $\tilde{A}_{\alpha j} = 0.$ Note that ${\tilde{A}}$ is a rectangular matrix: if there are *M* interparticle contacts and *N* particle displacements, then ${\tilde{A}}$ is an $M\times N$ matrix. As one approaches Point J, the number of contact forces decreases, being equal to *N* at the transition.

The spring exerts a restoring force that is proportional to this compression; the spring constant $k_\alpha$ can be different for each spring. The restoring force on each particle is the sum of the forces from all the springs it is connected to. Therefore,

2 $$\begin{aligned} m_j \ddot{x}_j = - {\tilde{A}}^T_{j\alpha } k_\alpha \Delta \ell _\alpha , \end{aligned}$$

i.e., the $\alpha$’th contact force acts as a restoring force on $x_j$ only if $x_j$ is associated with one of the two particles involved in the $\alpha$’th contact, in which case $A^T_{j\alpha }$ is the same projection factor $\cos \theta _{\alpha j}$ that we had in the dependence of $f_\alpha$ on $x_j.$ Putting this equation together with Eq. (1), we have

3 $$\begin{aligned} m_j \ddot{x}_j = - {\tilde{A}}^T_{j\alpha } k_\alpha {\tilde{A}}_{\alpha i} x_i. \end{aligned}$$

Defining $A_{\alpha j} = \sqrt{k}_\alpha {\tilde{A}}_{\alpha j}/\sqrt{m}_j,$ this is equivalent to

4 $$\begin{aligned} \sqrt{m_j}\ddot{x}_j = - A^T_{j\alpha } A_{\alpha i} \sqrt{m_i} x_i \end{aligned}$$

We have implicitly assumed that the particles are frictionless spheres, so that torque balance is trivially satisfied. Absorbing a factor of $\sqrt{m_j}$ in $x_j$ for each *j*,  we finally have the equation

5 $$\begin{aligned} \ddot{x}_j = - A^T_{j\alpha } A_{\alpha i} x_i , \end{aligned}$$

i.e., the dynamical matrix *K* is a $N\times N$ matrix that is equal to $A^T A.$

In the random matrix approach to this problem, we assume that all the entries in the matrix *A* are independent Gaussian random variables, drawn from a distribution with zero mean and (with a suitable rescaling) unit variance. This is the Laguerre random matrix ensemble (also known as the Wishart ensemble in the mathematical literature), and is different from assuming that the elements of *K* are independent Gaussian random variables (with the constraint that *K* is a symmetric matrix). The approach to Point J is modeled by adjusting the ratio *M*/*N* to approach 1 from above.

The random matrix approach ignores the fact that each contact force acts between only two particles, i.e., that each row in the matrix *A* has only 2*d* entries. It also ignores the fact that the network of contacts is created by particles rearranging themselves, which would be expected to result in correlations between the contact forces. To address these limitations, one would need to construct a model which includes spatial information about the contacts (or perform a full molecular dynamics simulation), discussed in Sect. 4 of this paper. When this is done, the non-universal results from random matrix theory are affected without changing the universal results.

It can be shown [37] that, for $M \ge N,$ the density of states for the eigenfrequencies $\omega _1, \omega _2\ldots \omega _N$ is

6 $$\begin{aligned} \rho (\omega ){} & {} = \frac{1}{N \pi } \frac{\sqrt{(N b^2 - \omega ^2)(\omega ^2 - N a^2)}}{\omega } \nonumber \\{} & {} \quad a \sqrt{N}< \omega < b\sqrt{N} \end{aligned}$$

where $a = \sqrt{M/N}-1$ and $b = \sqrt{M/N} + 1.$ When $M/N > 1,$ there is a broad peak in $\rho (\omega ),$ with a gap in the spectrum near $\omega = 0.$ The peak is not symmetric, falling off much more sharply on the small $\omega$ side than on the large $\omega$ side. In the middle, the peak slopes downwards as $\omega$ is increased. As *M*/*N* is reduced, the gap shrinks while the width of the peak remains constant. When $M/N = 1,$ $\rho (\omega ) = \sqrt{4 N - \omega ^2}/(N\pi )$ which matches the Wigner semicircle law for the Gaussian orthogonal ensemble, and $\rho (\omega =0)\ne 0.$

**Fig. 1:**
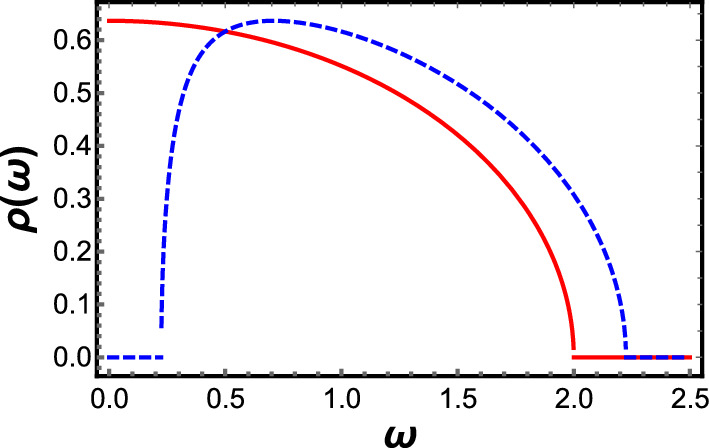
Plot of $\rho (\omega )$ vs $\omega ,$ with $\omega$ scaled by $\sqrt{N},$ for (red, solid) $M/N = 1.0$ and (blue, dashed) $M/N = 1.2.$ For $M/N = 1.2,$ the gap at small $\omega$ and the asymmetry of the peak are clearly visible. As *M*/*N* is increased, the asymmetry in the shape persists, the gap at small $\omega$ broadens, and the range of $\omega /\sqrt{N}$ for which $\rho (\omega ) \ne 0$ remains equal to $b-a = 2$

One can compare these analytical predictions with numerical results, with data from the O’Hern group [38]. The details of how the numerical simulations were conducted are given in Ref. [38], but to summarize: approximately 1000 frictionless bidisperse disks (half large and half small) with a diameter ratio of 1.4 were placed randomly in a square cell with periodic boundary conditions, and allowed to relax to equilibrium, at which point the frequencies of the normal modes were measured. The interaction between the disks was harmonic (only under compression), but the spring constant was different for large–large, large–small and small–small contacts. As with the analytical prediction, there is a broad peak that falls off more sharply at small $\omega$ than at large $\omega .$ The density of states $\rho (\omega =0) = 0$ except at the transition. However, the numerical data does not show the gap in the spectrum near $\omega = 0$ predicted by random matrix theory. The numerical data also has a pronounced boson peak at a nonzero value of $\omega ,$ and a cusp in $\rho (\omega )$ at the origin at the transition [15]. These features do not agree with the prediction from random matrix theory, but as already discussed, random matrix theory is not expected to yield the (non-universal) density of states correctly. We return to this point in Sect. 4, on the random lattice model.

## Correlations

In this section, we subject the predictions of the random matrix model to more stringent and appropriate tests, by studying the (universal) correlations. The key feature of the random matrix spectrum is that it is rigid (i.e., highly correlated). The rigidity of the spectrum is revealed at small energy scales by the distribution of consecutive level spacings. The longer range rigidity can be demonstrated by the autocorrelation of the density of states or by specific statistical measures such as the number statistic and the spectral rigidity [20].

Insight into the strong correlations between the eigenvalues implied by the Laguerre ensemble distribution is provided by the following plasma analogy. We focus on the case $M = N+1$ since we are interested in the spectrum for point J. For the Laguerre ensemble, the rigidity of the spectrum arises from the factors of $|\omega _j^2 - \omega _i^2|$ in the joint probability distribution $p (\omega _1, \omega _2, \ldots , \omega _N)$ for the eigenvalues given by Eq. (A2) If we rewrite this as $\exp (\ln |\omega _j - \omega _i | + \ln |\omega _j + \omega _i |),$ we can interpret the probability distribution as the partition function of a classical plasma of N particles located on the positive $\omega$ axis at the locations $\omega _1, \ldots , \omega _N$ with logarithmic interactions between the particles as well and between each particle and image particles at locations $-\omega _1, \ldots , -\omega _N$. The particles are also confined near the origin by a quadratic potential and are constrained to remain on the positive $\omega$ axis by a hard wall at the origin. The plasma analogy is another argument to only consider correlations for $M\approx N.$ If $M/N > 1$ (in the $N\rightarrow \infty$ limit), the gap in the Laguerre spectrum $\rho (\omega )$ near $\omega = 0$ means that the particles and their images are well separated. The correlations between energy levels in the Laguerre ensemble will then be indistinguishable from those for the GOE. Even for $M\approx N,$ the difference between the two ensembles should be greatest near $\omega = 0.$

In terms of the variables $x_i = \omega _i^2$, the one-point and two-point correlation functions are defined as

7 $$\begin{aligned} R_1 (x) = N \int _0^\infty d x_2 \ldots \int _0^\infty d x_N P(x, x_2, \ldots , x_N)\nonumber \\ \end{aligned}$$

and

8 $$\begin{aligned}{} & {} R_2 (x, y) = N(N-1) \int _0^\infty d x_3 \ldots \nonumber \\{} & {} \quad \int _0^\infty d x_N P(x, y, x_3,\ldots , x_N) \end{aligned}$$

Here, *P* denotes the joint probability distribution of the squared frequencies $x_i = \omega _i^2$ which can easily be deduced from the joint probability distribution $p ( \omega _1, \omega _2, \ldots , \omega _N)$ given in Eq. (A2). The plasma analogy shows that calculation of the correlation functions in Eqs. (7) and (8) is a formidable problem in classical statistical mechanics. Nonetheless, it has been exactly done by Nagao and Slevin [25] by rewriting the probability density in the form of a quaternion determinant and performing the integrals by a generalization of a theorem of Dyson [39] on integration over quaternion determinants. Before we give those results, we first describe the unfolding procedure.

$R_1(x)$ is evidently the density of states, and we now define

9 $$\begin{aligned} \xi (x) = \int _0^x d x^\prime \; R_1 (x^\prime ) \end{aligned}$$

where $\xi (x)$ is the cumulative density of states. The unfolded two point correlation function is then defined as

10 $$\begin{aligned} L_2 (\xi _1, \xi _2) = \frac{1}{R_1[ x (\xi _1) ]} \frac{1}{R_1 [ x(\xi _2) ]} R_2 [ x (\xi _1), x(\xi _2)]\nonumber \\ \end{aligned}$$

The exact expression for $L_2$ is rather lengthy and is given in the Appendix.

**Fig. 2:**
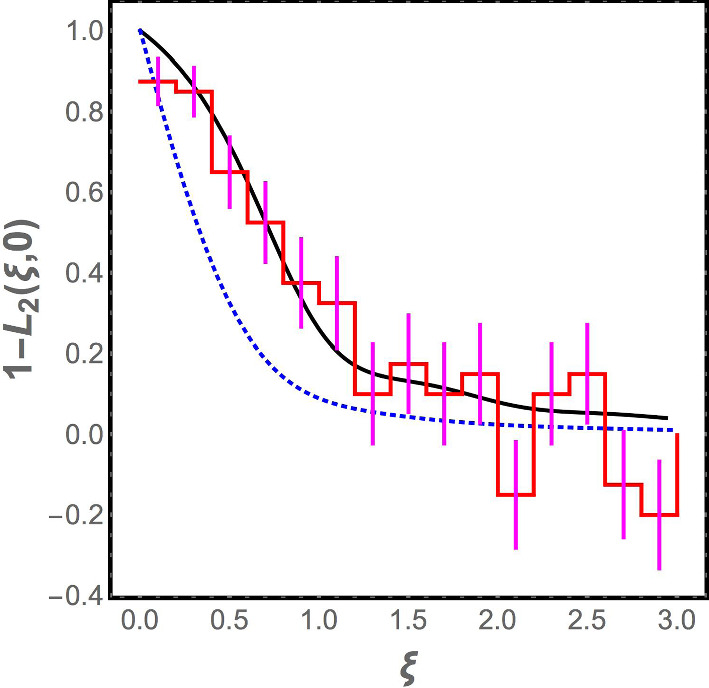
The correlation function $1 - L_2 (\xi , 0)$ is computed using vibrational spectra from 1000 realizations of the jammed state. It appears as a staircase in the plot due to the finite widths of the bins used to compute the correlation. Also shown are the analytic results for the Gaussian orthogonal ensemble (dotted blue curve) and the Laguerre ensemble (solid black curve). The agreement of the vibrational data to the Laguerre ensemble is clearly superior. The vibrational spectrum data are based on simulations by Kyle Vander Werf and Corey O’Hern described in Sect. 2

In Fig. 2, we plot $1 - L_2(\xi , 0)$ as a function of $\xi$ for the Laguerre ensemble. The corresponding plot for the Gaussian orthogonal ensemble is also shown. When $\xi$ is large, the two curves approach each other. Indeed, the analytical expression for $1 - L_2(\xi )$ for $\xi \gg 1$ in the Laguerre ensemble can be verified to be

11 $$\begin{aligned}{} & {} 1 - L_2 (\xi , 0)\nonumber \\ {}{} & {} = \frac{\sin ^2 \pi \xi }{ ( \pi \xi )^2} + \left[ \frac{1}{2} - \int _0^\xi d y \frac{ \sin \pi y}{\pi y} \right] \left[ \frac{ \cos \pi \xi }{\xi } - \frac{1}{\pi \xi ^2} \sin \pi \xi \right] \nonumber \\ \end{aligned}$$

which coincides with the form for the same quantity in the GOE, as expected since the effect of the image charges in the plasma analogy for the Laguerre ensemble should be small when $\xi$ is large. However, although the two curves coincide in the asymptotic limit $\xi \gg 1$, Fig. 2 shows that there is a range of $\xi$ values where the predictions of the Laguerre ensemble differ significantly from the GOE. Hence, comparison to the correlation function for the numerical data for the spectrum of the jammed bead pack provides a stringent test that is able to distinguish between the Laguerre ensemble and the GOE.

The numerical data are analyzed as follows. The vibrational frequencies obtained in the numerical simulations from all the 1000 realizations of the jammed state are merged together, and bins are constructed with 200 eigenvalues in each, i.e., there is an average of 0.2 eigenvalues per realization of the jammed state in each bin. Next, we calculate

12 $$\begin{aligned} 1 - \frac{1}{(0.2)^2} [\langle n_0 n_i\rangle - \langle n_0 \rangle \delta _{i0}] \end{aligned}$$

where $n_i$ is the number of vibrational frequencies in the $i^{\textrm{th}}$ bin in any given realization, and the average is over the realizations of the jammed state. The histogram of the values obtained for this discretized correlation function is compared with the analytical prediction from the Laguerre ensemble and the GOE, and as seen in Fig. 2, the Laguerre ensemble fits the data very well within the error bars (while the GOE does not).

**Fig. 3:**
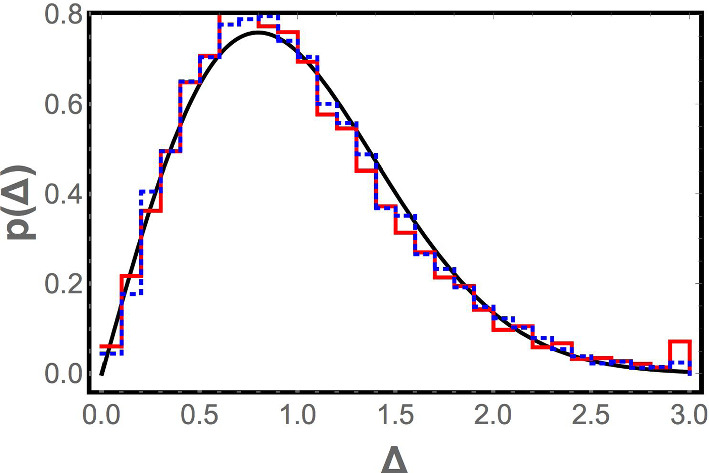
The red (solid) and blue (dashed) histograms show the consecutive level spacing distribution for the numerically computed vibrational spectra of jammed frictionless granular material. An ensemble of one thousand realizations of the jammed state was used. The red histogram bins the eleven consecutive level spacings between the frequencies $\omega _5$ through $\omega _{16}$ for each realization; the blue histogram eleven consecutive spacings between frequencies $\omega _{400}$ through $\omega _{411}$. Each spacing $\omega _{i+1} - \omega _i$ is normalized by $\langle \omega _{i+1} - \omega _i\rangle ,$ where the average is taken over the one thousand realizations. The black curve corresponds to the Wigner surmise for the level spacing distribution of the Gaussian orthogonal ensemble (which is indistinguishable from the Wigner surmise for the Laguerre ensemble at this level of resolution, as discussed in Appendix Appendix B). The close agreement between the two histograms and the solid black curve are consistent with the predictions of our random matrix model of the jammed state of frictionless granular matter

For completeness, we also compute the distribution of level spacings from the numerically computed vibrational spectra for bead packs [38]. This is shown in Fig. 3. Although the plasma analogy would suggest that the level spacing distribution could be different in the vicinity of $\omega = 0$ and for large $\omega ,$ this is not found to be the case; both distributions are found to be indistinguishable from the Wigner surmise, which also matches—within our level of resolution— the distributions for the Laguerre ensemble [24] and the GOE. Thus, although the level spacing distribution is consistent with the Laguerre ensemble, it does not discriminate between the Laguerre and Gaussian orthogonal ensembles.

## Random Lattice model

As discussed earlier, the extent to which the density $\rho (\omega )$ of vibrational frequencies for jammed frictionless granular materials agrees with the predictions of random matrix theory is not a good test of the applicability of random matrix theory to these materials, because the distribution of eigenvalues is a non-universal prediction of random matrix theory. Nevertheless, there are qualitative discrepancies between the numerically measured $\rho (\omega )$ [38] and the density of eigenvalues $\{\omega _i\}$ obtained from random matrix theory that are worth trying to address: the low-frequency gap, the absence of a cusp at $\omega = 0$ at the transition and the absence of a boson peak.

In Eq. (5), we have assumed that the entries in the matrix *A* are all independent random variables drawn from a Gaussian distribution. In reality, since the matrix *A* is supposed to be a mapping from coordinates to contact forces, and only two particles are associated with a contact, only the entries associated with two particles (with *d* entries per particle for *d*-dimensional particles) should be nonzero in any column of *A*. Thus, *A* should be a sparse matrix.

In mean field theory, one would choose the two particles associated with each force randomly. This would result in the system breaking up into separate clusters, not connected to each other, leading to an overabundance of zero modes unless one only retains the giant connected cluster. Moreover, the concept of adjacency would not be respected: two randomly chosen particles would be likely to be far apart, and should not have been allowed to share a contact.

**Fig. 4:**
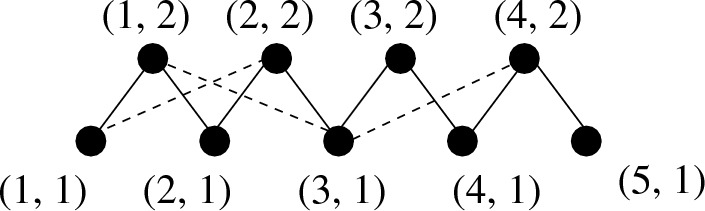
Arrangement of particles in the random lattice model. Each particle rests on at least the two nearest neighbors below it, shown as solid lines. In addition, a particle occasionally also has a contact with a next nearest neighbor below it on one side or the other. For clarity, these are shown as dashed lines, even though they are completely equivalent to the solid bonds. Each particle therefore has two, three or four contacts from above. Periodic boundary conditions are imposed on all four boundaries. As shown, the numbering convention results in the particle (*x*, *y*) being shifted horizontally by half a lattice spacing compared to $(x, y - 1).$ The bond angles are chosen randomly, and the vibrational spectrum calculated

Instead of choosing the particles associated with a force randomly, we approximate the system as being equivalent to a triangular lattice (with periodic boundary conditions), but with each particle displaced from the position where it would be in a perfect triangular lattice. This randomizes the orientation of the contacts between particles (Fig. 4).

To be specific, particles are arranged in successive horizontal layers, with each particle having contacts with the two particles immediately below it: slightly to the left and slightly to the right. Shifting the numbering in each row by half a lattice spacing relative to its predecessor, the particle (*i*, *j*) connects to the particles numbered $(i, j - 1)$ and $(i + 1, j - 1)$ with periodic boundary conditions in both directions. (A particle in the bottom layer, (*i*, 1),  connects with $(i - L/2, L)$ and $(i + 1 - L/2, L)$ in the topmost layer, where *L* is the number of layers.) In addition, each particle has a probability *p* of connecting to an additional particle in the row below it, i.e., a probability of 0.5*p* of connecting to the particle at $(i - 1, j - 1)$ and a probability of 0.5*p* of connecting to the particle at $(i + 2, j - 1),$ again with periodic boundary conditions. All contacts are bidirectional, so that each particle is connected to two to four particles in the row above it. The spring constant $\kappa$ associated with each contact is an independent random variable, with probability density

13 $$\begin{aligned} \rho (\kappa )d\kappa = 3 \kappa ^2 \exp [-\kappa ^3] d\kappa . \end{aligned}$$

This is what one would expect with exponentially distributed contact forces. Since the contact force *f* at a Hertzian contact is related to the compression $\delta x$ of the contact as $f\sim (\delta x)^{3/2},$ and the linear stiffness if the compression $\delta x$ is changed by a small amount is found by differentiating this expression to be $\kappa \sim (\delta x)^{1/2},$ therefore $f \sim \kappa ^3.$ We choose units in which $f = \kappa ^3.$ Thus, an exponential probability density for *f* implies

14 $$\begin{aligned} \rho (f) df = \exp [-f] df = \exp [-\kappa ^3] (3 \kappa ^2 d\kappa ) \end{aligned}$$

The bond connecting a particle to its left (right) neighbor in the row below points down and to the left (right), and the bond angles are uniform random variables in the third (fourth) quadrant. If the particle at (*i*, *j*) is also connected to $(i-1, j - 1),$ this bond angle is also chosen to be a uniform random variable in the third quadrant, with the constraint that it must be more horizontal than the bond between (*i*, *j*) and $(i, j - 1).$ (There is a similar condition if the additional contact is to the right, to $(i + 2, j - 1).$) This model is similar to a model introduced for free-standing granular piles [28], which is a vector generalization of the scalar-force ‘q-Model’ used to model such systems [1, 2]. We expect that our results will not depend on the details of the model, as long as the spring constants and bond angles are random, the locality of interactions is respected, and the number of contacts made by an individual particle can vary in order to approach the jamming transition.

Having constructed the lattice, it is easy to obtain the matrix *A*, and therefore the eigenvalues of $A^T A.$ One difference from the Laguerre ensemble is that each contact is associated with two particles, and therefore four displacement variables, i.e., each row of $A_{\alpha i}$ has exactly four nonzero entries. Another difference is that the translational invariance of the system is respected by the matrix *A*; the consequence of this is discussed later in this section.

Unlike the dynamical simulations, we compare with [38], the random lattice model uses a Hertzian contact force law rather than a harmonic law (with a distribution of spring constants). It also has the property that every lattice site is connected to at least two above it and two below, whereas granular assemblies will have some particles with less than four contacts. Most importantly, the random lattice model does not enforce force balance at each lattice site. The objective with the random lattice model is to show that randomness and locality are sufficient to yield the qualitative aspects of the density of states while preserving the level spacing distribution and the spectral correlations of Random Matrix Theory with the Laguerre ensemble. An improved version of the model, which would be a more complicated version of the model in Ref. [28]—because we do not have gravity to break the symmetry of the system, finding the equilibrium state is more complicated—can be studied if one is interested in how the shape and location of an eigenmode is related to force chains in the underlying stresses. We leave this for future work.

The random lattice model (RLM) with $128 \times 128$ sites was simulated in this manner, and the vibrational frequencies from 100 different realizations of randomness were merged and plotted as a histogram. The ratio of the number of contact forces to the number of coordinate degrees of freedom, which corresponds to *M*/*N*,  increases with the probability of a particle establishing extra bonds: from 1 at $p=0$ to 1.5 at $p=1.$

**Fig. 5:**
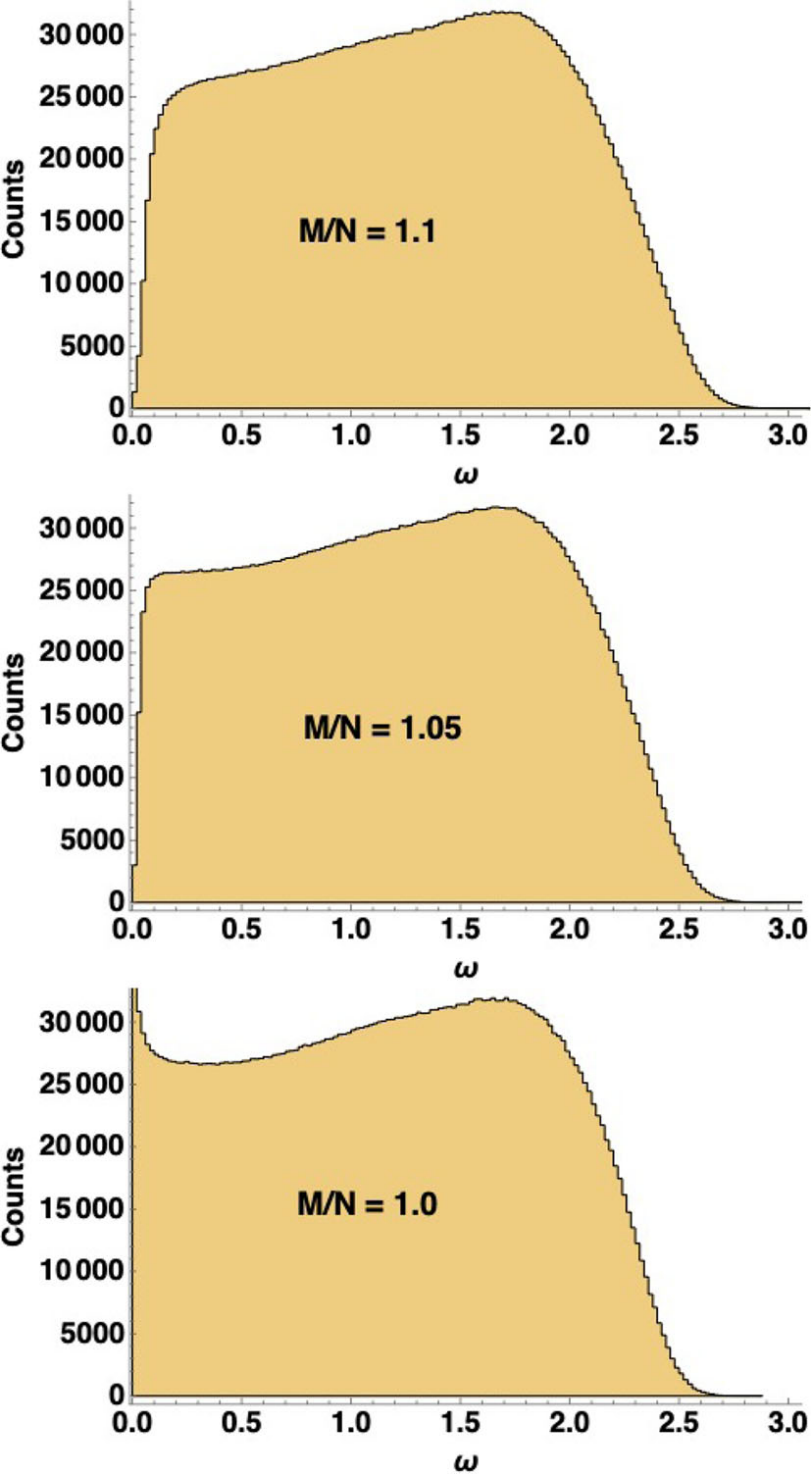
Histogram of vibrational frequencies obtained from the random lattice model described in this paper. One hundred realizations of 128x 128 random triangular lattices were constructed, with the ratio *M*/*N* equal to 1.1 (top), 1.05 (middle) and 1.0 (bottom) respectively

The results are shown in Fig. 5. When $M/N = 1,$ the density of states $\rho (\omega )$ is nonzero at $\omega = 0,$ in agreement with the dynamical simulations [38] and random matrix theory. But in addition, there is a cusp in $\rho (\omega = 0)$ at the transition point, as seen in the dynamical simulations but not in random matrix theory. (This cusp is also seen in some earlier mean field models [18, 27] which do not have local correlations; the random lattice model also has the advantage of being very similar to a model used successfully to explain force chains in static free-standing sandpiles.) The boson peak at $\omega \ne 0$ seen in the simulations is also reproduced for the lattice model, being most pronounced at the transition point.

Moving away from the transition point at $M/N = 1,$ there is a small gap in the spectrum of $\rho (\omega )$ near $\omega = 0.$ But this is a finite size effect; we have verified by increasing *N* while keeping *M*/*N* constant that the gap decreases. This is inevitable: the global translational invariance of the random lattice model with periodic boundary conditions ensures that there are two zero modes, and the locality of the connections that are made ensures that long wavelength oscillations have low frequencies. Thus, when $N\rightarrow \infty$ at constant *M*/*N*,  the density of states must scale as $\omega ^{d-1}$ for small $\omega .$ This is true in the dynamical simulations [38], and would be true for any local model, not just the random lattice model, but it is *not* true for random matrix theory as seen in Eq. (6) with fixed *M*/*N* and $N\rightarrow \infty .$

**Fig. 6:**
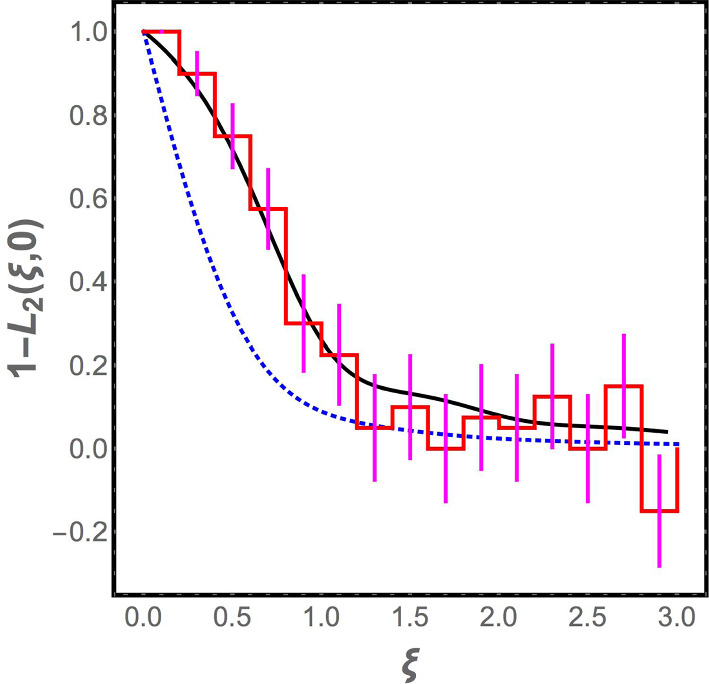
Correlation function $1 - L_2(\xi , 0)$ for the random lattice model introduced in this paper (red staircase) and for the Laguerre ensemble at the transition point (solid black curve). Good agreement is seen between the two. The correlation function for the Gaussian orthogonal ensemble is also shown for comparison (dotted blue curve)

**Fig. 7:**
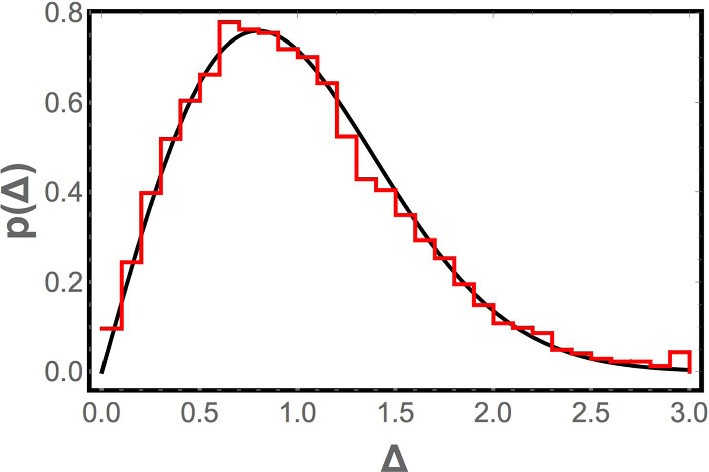
Histogram of level spacings for the random lattice model (red) and a fit to the Wigner surmise for the Gaussian orthogonal ensemble (solid black curve). Spacings from the fifth to the fifteenth normal mode frequencies are normalized, as discussed in the paper, and combined to create the histogram. The Wigner surmise fits the distribution very well, but as discussed in the paper, this applies equally to the Wigner surmise for the Laguerre ensemble

We see that the random lattice model reproduces the qualitative features of the numerical density of states $\rho (\omega )$—the cusp in $\rho (\omega )$ near the origin when $M/N = 1,$ the boson peak, the $\rho (\omega )\sim \omega ^{d-1}$ scaling when $M/N > 1$—that random matrix theory is unable to do. In addition, as seen in Fig. 6 and in Fig. 7, the distribution of spacings between consecutive frequencies is found to be the same as for the random matrix ensemble, consistent with the Wigner surmise, and the correlation function $1 - L_2(\xi ,0)$ at $M/N = 1$ matches that obtained for the Laguerre ensemble. (The correlation function is not compared away from the jamming transition because, as discussed earlier, it is indistinguishable from the GOE correlation function there.) This is what one would hope, since the system is in the universality class of the Laguerre ensemble rather than the GOE (since the dynamical matrix has the form $A^T A$), and any reasonable model of the system should be in the same universality class. Thus, it is reassuring that the random lattice model retains the positive features of random matrix theory, while curing its problems.

## Conclusions

In this paper, we show that a random matrix approach can be used successfully to calculate the correlations between vibrational frequencies in a granular system near the jamming transition, if the matrix ensemble is chosen correctly. By modifying the random matrices according to physical considerations, a random lattice model is constructed, which retains the correlation functions of random matrix theory and also successfully reproduces all the qualitative features in the density of vibrational frequencies. The random lattice model closely resembles a vector generalization of the *q*-model that has previously been used successfully to understand the distribution of stress in static granular matter [1, 2, 28]. That the same model is able to reproduce both the static and vibrational properties of granular matter suggests it may be more broadly applicable to provide a unified understanding of the physics of granular matter.

## Figures and Tables

**Fig. 8:**
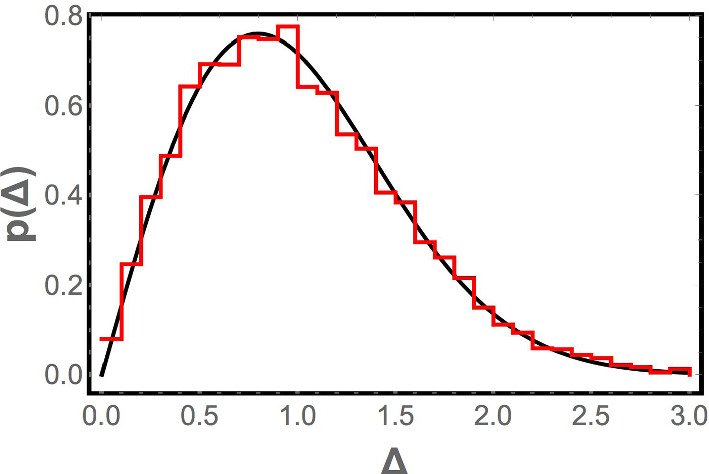
Histogram of the spacing $\omega _6 - \omega _5$, between the fifth and sixth frequencies for an ensemble of 10,000 spectra drawn from the Laguerre ensemble as described in the text. The spacings have been rescaled so that their mean value is one. The solid black curve is the Wigner surmise for the Gaussian orthogonal ensemble which is indistinguishable from the Wigner surmise for the Laguerre ensemble, at the resolution of this plot

**Fig. 9:**
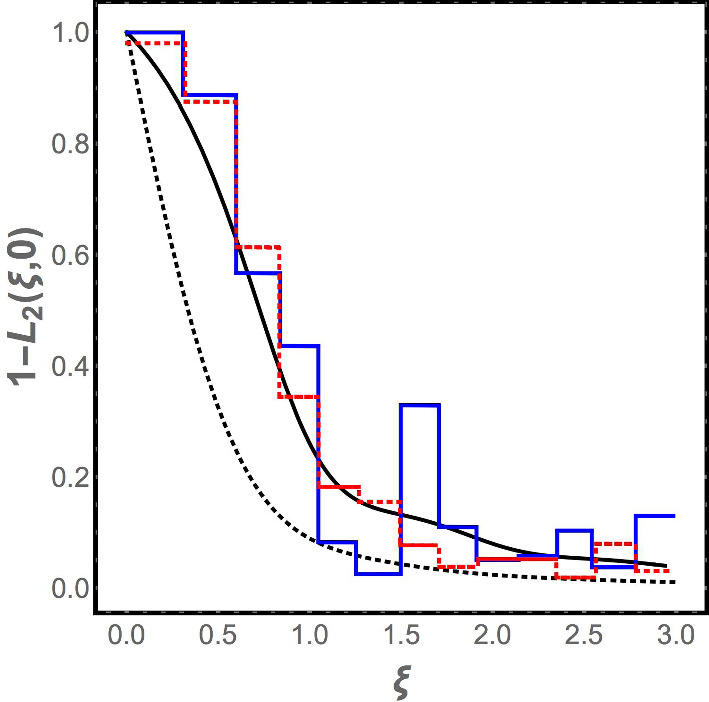
Plot of the correlation function for the Laguerre ensemble (solid curve) and the GOE (dotted curve). The blue (solid) histogram is the correlation function for an ensemble of 1000 spectra drawn from the Laguerre ensemble generated as described in the previous section. The red (dashed) histogram is the same but for a bigger ensemble of 10,000 spectra. Comparison of the two reveals that the departure of the numerical correlation from the exact analytic result is due in part to the finite bin width (which is the same in both cases) but in part also due to the finite size of the ensemble used to estimate the correlation. The exact analytic result for the Laguerre ensemble is shown as a smooth black curve; the dotted black curve is the correlation function for the Gaussian orthogonal ensemble

## Data Availability

The models developed in this paper were compared to numerical simulations of the vibrational spectrum of two-dimensional bead packs by the O’Hern group [38]. The numerical data are compared to model predictions in Figs. 1 and 2. Data are available from the authors upon reasonable request.

## Density of states

We start from the probability distribution for the $M\times N$ rectangular matrix *A*,  with each element of *A* an independent random Gaussian variable with zero mean and unit variance:

A1 $$\begin{aligned} p(\{A\}) \prod _{i, j} dA_{ij}\propto & {} \exp [- Tr(A^T A)/2] \prod _{ij} dA_{ij} \nonumber \\= & {} \prod _{ij} \left( \exp [- A_{ij}^2/2] dA_{ij}\right) . \end{aligned}$$

The Gaussian factor can be rewritten in terms of the eigenvalues $\omega _1^2, \omega _2^2\ldots \omega ^2_N$ of $A^T A$ as $\exp [-\sum \omega _i^2/2].$ Using the standard methods of random matrix theory, the measure $\prod _{ij} dA_{ij}$ can be expressed in terms of the $\omega _i$’s, and ‘angular variables’ which can be integrated out. The reduced probability density for the $\omega _i$’s is

A2 $$\begin{aligned} p(\{\omega \}) \propto \prod _{i < j} |\omega _j^2- \omega _i^2|\prod _i \omega _i^{M - N} \exp \left[ - \sum _i \omega _i^2/2\right] . \end{aligned}$$

Without loss of generality, we have chosen all the $\omega _i$’s to be greater than zero. The distribution in Eq. (A2) is known as the Marchenko–Pastur distribution in the mathematical literature.

Rewriting the probability density in terms of $\lambda _i = \omega _i/\sqrt{N},$ we have

A3 $$\begin{aligned} p(\{\lambda \}) \propto \exp \left[ - N \sum _i \frac{\lambda _i^2}{2} + f \ln |\lambda _i| + \sum _{i < j} \ln |\lambda _i^2 - \lambda _j^2|\right] \end{aligned}$$

where $M/N = 1 + f.$ If $M - N$ is *O*(*N*),  a saddle-point expansion yields

A4 $$\begin{aligned} \frac{f}{\lambda } - \lambda + P\int _0^\infty \rho (\lambda ^\prime )\left[ \frac{1}{\lambda - \lambda ^\prime } + \frac{1}{\lambda + \lambda ^\prime }\right] d\lambda ^\prime \end{aligned}$$

wherever $\rho (\lambda ) \ne 0.$ Here $\rho (\lambda )$ is the density of eigenvalues, normalized to $\int _0^\infty \rho (\lambda ) d\lambda = 1,$ and the *P* denotes the principal value of the integral. Symmetrizing $\rho (\lambda )$ by defining $\rho (\lambda < 0) = \rho (-\lambda ),$ the function

A5 $$\begin{aligned} F(\lambda ) = \int _{-\infty }^\infty \frac{\rho (\lambda ^\prime )}{\lambda - \lambda ^\prime } d\lambda ^\prime \end{aligned}$$

of the complex variable $\lambda$ is analytic everywhere except that it has branch cuts on the real line over intervals where $\rho (\lambda )\ne 0,$ where it is equal to

A6 $$\begin{aligned} \lambda - \frac{f}{\lambda } \mp i \pi \rho (\lambda ). \end{aligned}$$

Furthermore, $F(\lambda \rightarrow 0)$ is finite, and $F(\lambda \rightarrow \infty ) \rightarrow 2/\lambda ,$ because the symmetrized extension of $\rho (\lambda )$ integrates to 2. This has the solution

A7 $$\begin{aligned} F(\lambda ) = \lambda - \frac{f}{\lambda } - \frac{\sqrt{(\lambda ^2 - a^2) (\lambda ^2 - b^2)}}{\lambda } \end{aligned}$$

with

A8 $$\begin{aligned} a= & {} \sqrt{M/N} - 1\nonumber \\ b= & {} \sqrt{M/N} + 1. \end{aligned}$$

The density of states is then

A9 $$\begin{aligned} \rho (\lambda ) = \frac{1}{\pi } \frac{\sqrt{(b^2 - \lambda ^2)(\lambda ^2 - a^2)}}{\lambda } \qquad a< \lambda < b \end{aligned}$$

where we have removed the extension of $\rho (\lambda )$ to $\lambda < 0,$ so that $\int _0^\infty \rho (\lambda ) d\lambda = 1.$ The density of states $\rho (\omega )$ has the same form, but with *a* and *b* rescaled, which is not significant since Eq. (A2) was already obtained after rescaling.

## Level spacing distribution

In this section, we argue that the distribution of consecutive level spacings for the Laguerre ensemble Eq. (A2) is indistinguishable from the GOE. To this end, it is useful to recall that for the GOE, Wigner showed that a very good approximation to the level spacing distribution can be obtained by considering a model with just two levels. This formula, known as the Wigner surmise, is indistinguishable from the exact result, except in the tails of the distribution where, in any case, the weight is negligible, making the distinction irrelevant for applications. Intuitively Wigner’s surmise works because at small spacing the distribution is dominated by the interaction between the two consecutive levels; the other levels that are neglected in the analysis only matter at large spacings. In the same spirit, we consider the distribution Eq. (A2) for the case $M = N = 2$. We define $\Delta = \omega _1 - \omega _2$ as the spacing and $\omega = \frac{1}{2} (\omega _1 + \omega _2)$ as the mean energy of the two levels, and we take $\omega _1 > \omega _2$. For a fixed $\omega$, the level spacing distribution is then given by

B1 $$\begin{aligned} p ( \Delta ) \propto \Delta \exp \left[ - \frac{1}{4} \Delta ^2 \right] . \end{aligned}$$

for $0< \Delta < 2 \omega$ and $p = 0$ for $\Delta > 2 \omega$. The normalization factor is easily determined by imposing

B2 $$\begin{aligned}{} & {} N(\omega ) \int _0^{2 \omega } d \Delta \; \Delta \exp \left( - \frac{\Delta ^2}{4} \right) = 1 \nonumber \\{} & {} \quad \Rightarrow N( \omega ) = \frac{1}{2 [ 1 - \exp ( - \omega ^2 ) ]}. \end{aligned}$$

It is possible to work out $\overline{\Delta }(\omega )$ the mean value of $\Delta$ in closed form. We obtain

B3 $$\begin{aligned} \overline{\Delta } (\omega )= & {} \int _0^{2 \omega } d \Delta \; \Delta p (\Delta ) \nonumber \\= & {} \frac{\sqrt{\pi } \; {{\,\textrm{Erf}\,}}(\omega ) - 2 \omega \exp ( - \omega ^2) }{1 - \exp ( - \omega ^2 )} \end{aligned}$$

We now define a rescaled level spacing $\delta = \Delta / \overline{\Delta (\omega )}$ which has mean unity. The probability density function $\rho$ for $\delta$ is given by

B4 $$\begin{aligned} \rho ( \delta ) = \frac{ [ \overline{\Delta } (\omega ) ]^2 }{2 [ 1 - \exp ( - \omega ^2 ) ] } \delta \exp \left[ - \frac{ \overline{ \Delta }^2}{4} \delta ^2 \right] \end{aligned}$$

for $0 \le \delta \le 2 \omega / \overline{\Delta }$ and $\rho (\delta ) = 0$ for $\delta > 2 \omega / \overline{\Delta }$.

The parameter $\omega$ is a measure of how close the two consecutive levels are to the edge; to be precise $\int _0^\omega d \Omega R_1 (\Omega )$ is the ordinal number of the pair whose spacing distribution is being considered; here, $R_1$ is the mean density of states corresponding to the distribution in Eq. (A2). It is easy to verify that the distribution approaches the Gaussian orthogonal ensemble as $\omega \rightarrow \infty$. More surprisingly and perhaps disappointingly we find that for pairs of levels that are quite near to zero frequency also the Gaussian orthogonal ensemble is a good approximation. This means that in testing whether the spectrum of jammed granular matter is described by random matrix theory, we cannot use the level spacing to distinguish between our model and the Gaussian orthogonal ensemble.

Because the Wigner surmise is not exact, we have tested it by directly simulating the Laguerre random matrix ensemble. We generated an ensemble of ten thousand $M \times N$ random matrices *A* with $M = N = 100$. The matrix elements were drawn from a Gaussian distribution with zero mean and unit variance. We then evaluated the eigenvalues, $\omega _i^2$ of $A^T A$. A histogram of the spacing between the fifth and sixth levels, $\omega _6 - \omega _5$, in the ten thousand different realizations, was plotted (see Fig. 8). The spacings were scaled to have unit mean. The Wigner surmise for the Gaussian orthogonal ensemble (and the Laguerre ensemble) was found to be an excellent fit to the numerical data, confirming the analysis above.

It is possible to derive an exact expression for the level spacing distribution using the technology of quaternion determinants developed by Dyson [39] and Mehta [20] and generalized to the Laguerre ensemble by Nagao and Slevin [25]. This analysis would allow a more careful study of the tails of the distribution where it might deviate from the simple approximation derived here. Such an analysis is not needed for the application considered in this paper but is of intrinsic mathematical interest and we will return to it elsewhere.

## Correlations of the Laguerre ensemble

Nagao and Slevin [25] have shown that the correlations for the Laguerre ensemble can be computed by rewriting the distribution as a quaternion determinant and performing the required integrals using a powerful generalization of a theorem by Dyson [39]. Here, we summarize their results, taking the opportunity to correct some typos in their paper, and to present the results in a form that does not require the reader to have familiarity with the specialized language of quaternion determinants or Pfaffians.

We start by defining the unfolding function

C1 $$\begin{aligned} \xi (x) = \frac{1}{2} \int _0^x d t \; \left[ t J_1^2 (t) - t J_0 (t) J_2 (t) + J_0(t) J_1 (t) \right] . \end{aligned}$$

Note that the order of the Bessel function in the first term of the integrand above is given incorrectly in Ref. [25].

Next we define

C2 $$\begin{aligned} r(x) = \sqrt{ J_1^2 (x) + \frac{1}{2} J_0^2 (x) - \frac{1}{2} J_0 (x) J_2 (x) } \end{aligned}$$

and

C3 $$\begin{aligned} \mathcal{C} (x, x^\prime ) = 2 \frac{x J_1 (x) J_0 (x^\prime ) - x^\prime J_0 (x) J_1 (x^\prime ) }{(x-x^\prime )(x + x^\prime )} - \frac{J_0(x) J_1(x^\prime )}{x^\prime }. \end{aligned}$$

In terms of these functions, one can write down

C4 $$\begin{aligned} S (\xi , \xi ^\prime ) = \frac{1}{r(x) r(x^\prime )} \mathcal{C} (x, x^\prime ) \end{aligned}$$

where it is understood that *x* is short for $x(\xi )$ and $x^\prime$ for $x (\xi ^\prime )$, the inverse of the function given by Eq. (C1). We also write

C5 $$\begin{aligned} I (\xi , \xi ^\prime ) = \frac{1}{r(x) r(x^\prime )} \left[ \int _{x^\prime }^x d t \; \frac{t}{2} \mathcal{C}(x, t) - \theta (x - x^\prime ) + \frac{1}{2} \right] \end{aligned}$$

where $\theta$ denotes the unit step function and

C6 $$\begin{aligned} D (\xi , \xi ^\prime ) = \frac{2}{ x r(x) r(x^\prime )} \frac{\partial }{\partial x} \mathcal{C}(x, x^\prime ); \end{aligned}$$

the variable of integration and the arguments of the integrand in Eq. (C5) are given incorrectly in Ref. [25] (Fig. 9).

With these definitions established, we can now write down the principal result of Nagao and Slevin [25], namely

C7 $$\begin{aligned} 1 - L_2 (\xi _1, \xi _2 )= & {} S (\xi _1, \xi _2) S ( \xi _2, \xi _1 ) + \frac{1}{2} I (\xi _1, \xi _2 ) D ( \xi _2, \xi _1 ) \nonumber \\{} & {} + \frac{1}{2} I (\xi _2, \xi _1 ) D (\xi _1,\xi _2 ). \end{aligned}$$

We are interested in $1 - L_2 (\xi , 0)$. For this special case, we obtain the simplifications

C8 $$\begin{aligned} S(\xi , 0 )= & {} \frac{\sqrt{2}}{r(x)}\left[ \frac{1}{2} J_0 (x) + J_2 (x) \right] , \nonumber \\ S( 0, \xi )= & {} \frac{\sqrt{2}}{r(x)} \left[ \frac{1}{2} J_0 (x) + \frac{1}{2} J_2 (x) \right] , \nonumber \\ I (\xi , 0)= & {} \frac{\sqrt{2}}{r(x)} \left[ \int _0^x dt\; \frac{t}{2} \mathcal{C} (x, t) - \frac{1}{2} \right] , \nonumber \\ I (0, \xi )= & {} \frac{\sqrt{2}}{r(x)} \left[ \frac{1}{2} J_0 (x)\right] ,\nonumber \\ D (\xi , 0)= & {} - \frac{\sqrt{2}}{r(x)} \left[ \frac{1}{6} J_2 (x) + \frac{1}{6} J_4 (x) \right] , \nonumber \\ \rho ( 0, \xi )= & {} - \rho (\xi , 0 ). \end{aligned}$$

Equations (C7) and (C8) are the principal results needed for our test of the random matrix model. As shown in this paper, the histogram of the vibrational frequencies from the numerical simulations agrees with the correlation function for the Laguerre ensemble, but not the Gaussian orthogonal ensemble (Fig. 9). The deviation from the histogram for large $\xi$ is consistent with the error bars, and is also consistent with the deviations that are seen if a histogram is obtained by simulating the Laguerre ensemble 1000 times.
